# Portable Detection of Monkeypox virus Clades using Bridging LAMP

**DOI:** 10.21203/rs.3.rs-9014850/v1

**Published:** 2026-05-12

**Authors:** Mark D. Knappenberger, Siril Arockiam, Vi Nguyen, Karen Kibler, Mateusz Szczerba, James Bonner, Whitni Davidson, Kimberly Wilkins, Michael B. Townsend, Crystal Gigante, Christina L. Hutson, Yu Li, Panayampalli S. Satheshkumar, Jennifer Blain Christen, Bertram Jacobs, Karen S. Anderson

**Affiliations:** 1 Biodesign Center for Personalized Diagnostic; 2 School of Life Sciences; 3 School of Electrical, Computer, and Energy Engineering; 4 ASU-Banner Neurodegenerative Disease Research Center; 5 Centers for Disease Control; 6 Biodesign Center for Mechanisms of Evolution

## Abstract

*Orthopoxvirus monkeypox* (MPXV) is a zoonotic pathogen with two major clades that cause outbreaks in humans. Differentiation between the historically more clinically severe clade I and clade IIb is needed for effective public health response, such as in the ongoing 2022-2025 MPXV multi-country outbreaks. We have developed three loop-mediated isothermal amplification (LAMP) assays that differentiate between clade I, clade IIa/IIb MPXV, and broader *Orthopoxvirus* species by exploiting viral genomic rearrangements and amplicon length limitations inherent to LAMP. We demonstrate clade specificity between clade I and clade II viral cell culture lysates with LODs of 81-325 genomic copies per reaction and rapid detection of crude lesion specimens isolated from 45 patients during the latest MPXV USA 2022-2025 outbreaks. MPXV genomic DNA can be detected in as little as 10 minutes and clade-specific results detected in under an hour with limited instrumentation. We adapt a portable microfluidic system for point-of-need outbreak responses to zoonotic orthopoxvirus.

## Introduction

The recent emergence of orthopoxvirus monkeypox (MPXV) as a global viral threat has demonstrated the challenges of endemic virus control. Mpox (formerly monkeypox) is caused by two distinct clades of the dsDNA enveloped virus MPXV, which have been identified as endemic on the African continent since 1970 with historic human mortality rates approaching 1% and 3.5%^1^ for West African (clade II) and Congo Basin-derived strains (clade I), respectively. While historically clade I mortality rates have been thought closer to 10%, recent studies have shown that with modern care clade I survival increases significantly^2,3^. Sporadic outbreaks of clade II infection in traditionally MPXV-naive countries have seen isolated increases in frequency beginning with the US outbreak in 2003^4-7^, correlating with outbreaks overseas. Waning cross-immunity after discontinuation of vaccination campaigns for variola virus (causative agent of smallpox) in 1980 (in some countries earlier) has been at least partially implicated in the increase in case reports^2^; 70% of the world’s population is now estimated to be vulnerable to infection by orthopoxviruses (OPXV). Most non-African mpox cases from 2003 to 2021 were due to importation of clade II by infected travelers, however recently clade I MPXV cases have also spread outside of endemic regions^8,9^. Importation of exotic rodents, which are thought to constitute part of the poorly defined zoonotic reservoir of MPXV, have also been implicated in cross-country spread^2^.

In contrast, the Democratic Republic of Congo in which clade I is endemic has experienced both presumably zoonotic spillover events of clade I and documented sustained human-to-human transmission primarily due to sexual interactions, including around 60,000 suspected cases since 2024^10,11^. True mpox case burden is difficult to quantitate absolutely, however, due to low rates of laboratory confirmation. The current increase in worldwide cases outside of endemic areas and the difficulties in differentiating mpox from other rash causing illnesses within these areas^12^ necessitate tools for rapid clinical detection and differentiation.

Clinical clade differentiation requires specialized MPXV assays with high mismatch sensitivity or genetic sequencing approaches. The outbreak strain of the 2022 global mpox outbreak (clade IIb / USA2022_MA001) is derived from clade II which shares 99% sequence homology with clade I, however sustained human-to-human transmission is currently ongoing with at least four MPXV strains (clade IIb A.2.2., IIb lineage B, clade Ib, and clade Ia) showing the need for viral surveillance^13^. Many OPXVs lineages demonstrating broad host tropism are also endemic to each continent and maintain approximately 90% sequence homology with currently circulating MPXV clades. Thus, OPXVs and clinically distinct MPXV lineages pose challenges to developing clade-specific nucleic acid diagnostics.

Furthermore, both ends of the 200 kbp MPXV genome contain identical, mirrored 6.4 kbp repeats constituted by telomeric repeats and short tandem repeats^14^. Most of the 190 open reading frames of appreciable length (>180 nucleotides) are conserved between MPXV and OPXVs and coding region deletions are highly correlated to limitations in host tropism^2,14,15^. Thus, most current MPXV and OPXV diagnostics rely on probe-based detection of central, conserved genomic core regions or exploit genes with lineage-specific SNPs such as DNA polymerase (E9L) or viral envelope (B6R) to differentiate between clades^4,16-18^.

RT-qPCR-based methods currently recommended by the World Health Organization support the aim of ensuring sensitive and specific (i.e. accurate) tests are utilized; however they are poorly suited for point-of-care (PoC) implementation due to the need for thermocycling instrumentation, a constant power supply, and specially trained laboratory staff using dedicated laboratory space due to high risk of cross contamination. Although isothermal approaches have been developed for PoC settings^17,19-21^, these approaches require either enzymatic processing of amplification products for clade specificity and differentiation from common OPXVs or rely on proprietary technologies with expensive reagents.

Loop-mediated Amplification (LAMP) is an isothermal detection method amenable to the PoC detection of pathogens from biofluids. Microfluidic cartridge-based implementations of LAMP assays can be simple to use by minimally trained personnel, require minimal instrumentation (including colorimetric detection by eye), and have similar lower limits of detection (LLOD) to RT-qPCR at 10-100 target copies per reaction^22,23^. LAMP primer designs, however, typically have difficulty distinguishing highly homologous targets based on SNPs due to their length and the characteristics of *Bst* DNA polymerase. Secondary detection steps using Cas12b have been developed to increase specificity to single base pair resolution^24,25^, however the need to design an efficient protospacer into the amplicon reduces the already limited number of LAMP primer sets. Secondary hybridization probes likewise increase assay complexity, reagent cost, and decrease signal intensity compared to intercalating dye-based detection.

In this work we present a novel LAMP-based approach for detection and differentiation of clade I and II MPXV based on clade-specific genomic rearrangements and the kinetic limitations of Bst DNA Polymerase (Bridging LAMP). We demonstrate 100% concordance of our 3-plex assay format against benchmarked qPCR values for 80 skin lesion swab samples (50 positive, 30 negative) collected from patients presenting during the 2022 US outbreak (clade IIb). Additionally, we validated the clade specificity by differentiating between clade I and clade II templates utilizing cell culture lysates of Zaire-79 (clade Ia) and WRAIR 7-61 (clade IIa) strains. We determine lower limits of detection (LLOD) for these assays and define the linear range and precision of each assay across a broad range of dilution factors. We perform nanopore sequencing of both specific and non-specific background products and thus quantify the specificity of our clade II-specific approach challenged with clade I template. Detection of MPXV in 5 positive patient samples is demonstrated using a parallel 4-plex portable microfluidic assay. Finally, we detected 6/16 non-MPXV OPXVs to demonstrate the broad applicability of the pan-OPXV assay developed above.

## Results

### Rapid detection and discrimination of clade I, clade II MPXV, and broader OPXV *via* Bridging LAMP

**Figure 1.**
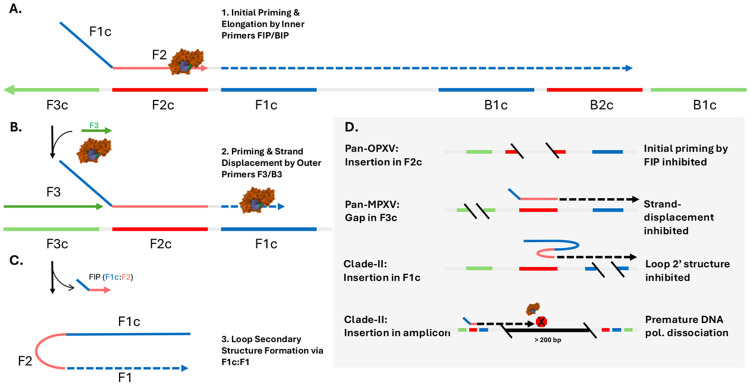
One-sided diagram of LAMP steps and four priming disruption strategies for the purpose of specific detection between highly homologous viral lineages. (A) Forward Inner Primer (FIP) 3’ end (F2) binds F2c on viral genome and undergoes strand extension up to 300 nts. F1c at 5’ of primer remains unbound. (B) Strand-displacement outer primer (F3) binds genomic F3c and is extended, displacing the extended FIP primer. (C) Displaced extended FIP product undergoes conformational change to stem loop structure via basepairing between F1c and newly synthesized F1. (D) LAMP assay strategies to confer specificity based on insertion in each target region.

**Figure 2.**
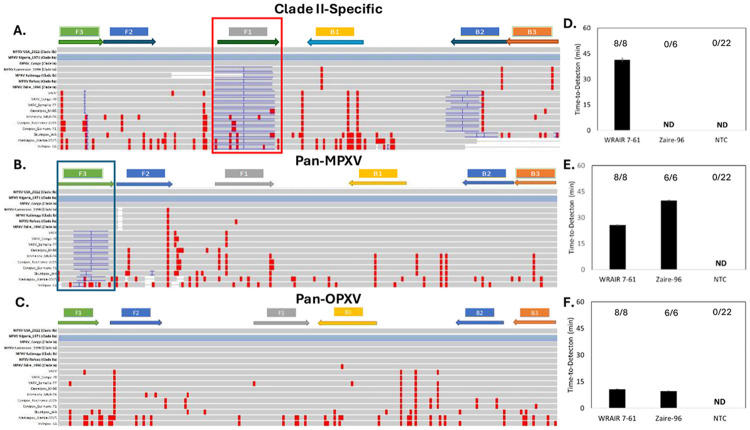
MPXV/OPXV alignments generated from NCBI nBLAST and validation of primer specificity. Primers for **(A)** clade II-specific targeting with sequence insertions (blue) overlapping the F1 and/or B2 target regions (red box) of clade I and OPXV alignments, **(B)** pan-MPXV primers with gap located at 5’ of F3 primer (blue box) in all OPXVs except Alaskapox which contains an insertion. Green boundaries represent end of amplicon alignment region. **(C)** pan-OPXV primers with regions of highest sequence homology targeted with inner and outer primers. **(D)** Time-to-detection (TTD) for viral cell culture lysate containing 10,000 copies clade II (WRAIR 7-61) vgDNA, clade I (Zaire-96) vgDNA, or no template control (NTC) for clade II-specific primers, **(D)** pan-MPXV primers, and **(E)** pan-OPXV primers. Number of reaction replicates listed for each experiment with an assay time cutoff of 60 minutes.

LAMP depends on two core primer pairs and DNA polymerase-catalyzed strand displacement to transition between initial priming by FIP and BIP (Figure 1A), inner primer extension, inner product displacement by F3 and B3 (Figure 1B), and amplicon secondary structure formation (Figure 1C). These preliminary steps result in the formation of a canonical double stem-loop template molecule (“dumbbell”) used for further logarithmic amplification through FIP and BIP binding to breathing dsDNA as well as loop primers that bind the ssDNA loops on each end. Disruption of any step *via* positioning of mutations and indels within orthologous primer target regions are the primary means for conferring target specificity to LAMP reactions (Figure 1D). Due to a lack of LAMP-targetable variations between MPXV clades I and II, we exploited a 289 base pair insertion common to clade I and broader OPXVs identified via alignment of six MPXV and eleven OPXV isolates (Figure 2A). The eleven OPXV isolates (spanning 8 host species) chosen were selected based on their prevalence on each continent, and mean sequence distance to clinically relevant MPXV clades. For the clade II-specific assay, the clade I/OPXV-specific translocation was positioned within the F1 primer target sequence (Figure 2A, **red box**) to inhibit dumbbell secondary structure formation and raise the amplicon size above the processivity limitations of *Bst* DNA polymerase— about 250 nucleotides^26^. To design the pan-MPXV assay without broader detection of OPXVs, a 13-nucleotide deletion specific to New World OPXVs bounded within the F3 primer region was used to inhibit displacement of inner primer extension products (Figure 2B, **blue box**). Lastly, the pan-OPXV assay was designed around a region of high sequence homology across MPXV and the New World Poxviruses (Figure 2C). The predicted specificities and hypothesized mechanisms of specific detection for each primer set are summarized in Table 1 and were quantified *in silico* via alignment of primers to each MPXV/OPXV by BLAST with manual mutation counting.

After identification of MPXV and OPXV-specific targets, we first analyzed primer secondary structure and probable dimerization products *in silico*. Primers identified as high-risk for non-specific amplification in the absence of target were ruled out primarily based on predicted secondary structure and primer heterodimer free energy at 65° C. We next empirically tested primer sets for non-specific amplification in an inert background and in the presence of off-target nucleic acid including human genomic DNA isolated from peripheral mononuclear cells and Varicella-Zoster Virus (VZV) BACMID DNA. Real-time fluorescence measurement (n=22 reactions per primer set) of SYTO9 intercalation of non-specific products after 60 minutes of amplification were used to categorize the non-specific nature of each set as (1) constant signal (No-BG) (2) linear increase in signal (lin-BG), (3) exponential increase in signal (log-BG), or (4) a combination of linear and exponential signal increase (linlog-BG) (Suppemental Figure S7). Out of six primer sets we initially predicted to have no background amplification, we categorized four as No-BG (pan-MPXV, pan-OPXV), one as lin-BG (clade II-specific MPXV), and one as log-BG (unnamed clade II-specific). We moved forward to testing specific template with the primer sets (Supplemental Table S1) exhibiting No-BG or lin-BG and low baseline (< 10% of measured fluorescence at plateau phase).

### Detection of clade I and II viral lysates

The performance of our clade II-specific assay was first tested by spiking in either WRAIR 7-61 (clade IIa) or Zaire-79 (clade Ia) viral genomic DNA isolated from cultured Vero Green Monkey cells directly to LAMP reactions. Amplification of samples with spiked-in clade II but not clade I lysate yielded positive signals (n=8) within 45 minutes (Figure 2D). Clade I lysate spiked samples showed no amplification after an hour of incubation at 65° C (n=8). Both strains amplified using the pan-MPXV primer set before 40 minutes at a dilution factor of 1:100 (Figure 2E). The pan-OPXV assay amplified both templates in under 12 minutes (Figure 2F).

**Figure 3.**
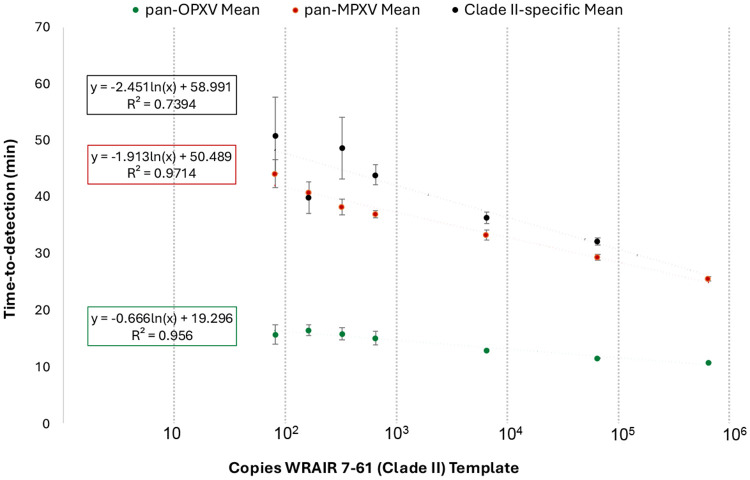
Detection of Clade II viral genomic DNA (from WRAIR 7-61 lysate) with clade II-specific, pan-MPXV, and pan-OPXV primer sets. Copies template versus time-to-detection values for 6 replicates per primer set. Error bars indicate standard deviation of the mean.

After validating clade specificity using culture lysates, we next determined lower limits of detection (LOD), time to detection, and assay precision over four orders of magnitude at the low end of the detectable range using serially diluted WRAIR 7-61 (clade IIa) (Figure 3). Assay LOD was determined as 4.1 clade II genomic copies per μL (81.3 cps/rx) for the pan-MPXV assay, 16.25 copies/μL (325 cps/rx) for clade II-specific assay, and 8.15 copies/μL (163 cps/rx) for pan-OPXV. Assay consistency was determined as time-to-detection variation for each primer set at each template copy number over 6 replicates.

### Patient swab samples from 2022 Clade IIb outbreak

**Figure 4.**
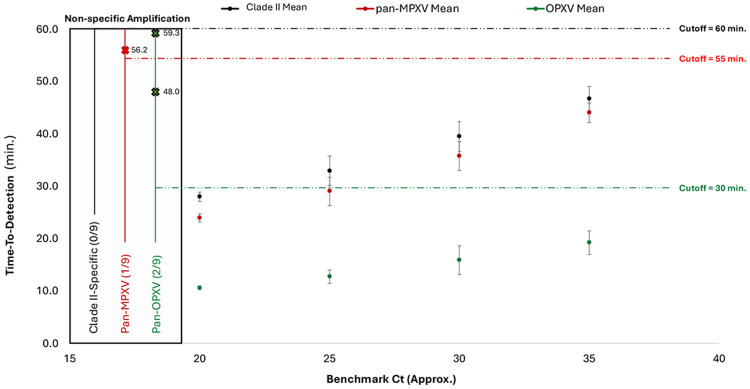
Sensitivity and specificity of each assay and combined assay format (2/3 or 3/3 parallel positive assays required to conclude positive) over entire CDC sample set.

The clade II, pan-MPXV, and pan-OPXV-specific assays were benchmarked to 80 crude lesion swab samples (50 positive, 30 negative) collected during the 2022 multi-country MPXV outbreak and previously characterized *via* clade II-specific and RNAse P qPCR. For each test, 2 μL of crude swab eluate was added directly to the LAMP reaction. Due to LAMP’s tolerance to inhibitors, we decided to test unpurified, heat-inactivated samples directly spiked into LAMP reactions in lieu of pre-purifying the viral genomic DNA. Mean time-to-detection was determined for each specimen from three independent replicates and plotted against benchmark Ct value (Figure 4), in addition to non-specific amplification controls with no template (n=9 for each primer set). Assays cutoff times were determined by selecting timepoints between the end of sample amplification and the start of non-specific amplification (Figure 4, **left inset**) which for the three primer sets (1) clade II, (2) pan-MPXV, and (3) OPXV were 60 minutes, 55 minutes, and 30 minutes, respectively. Our assays in a lab-based implementation successfully detected all 50 positive samples and, in a 3-plex format, began to yield positive signal in as little as ten minutes. The slowest amplification times noted were from the clade II-specific and pan-MPXV assays challenged with the Ct~35 samples, yielding a detectable signal at about 45 minutes.

**Figure 5.**
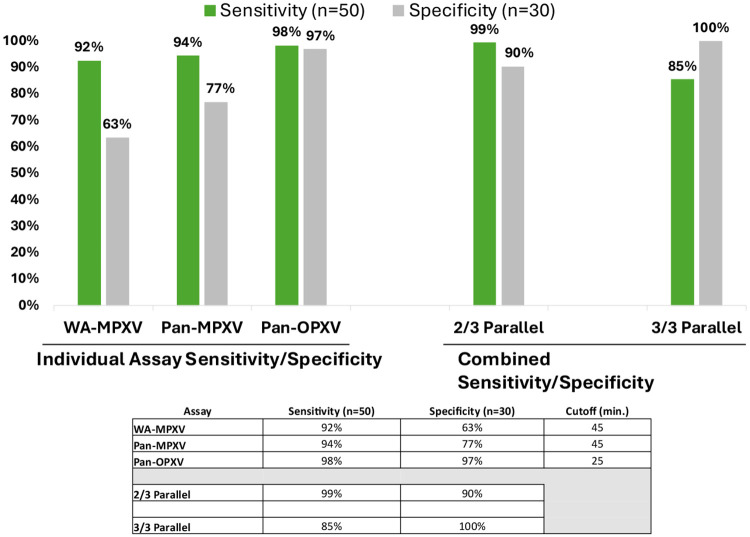
Combined sensitivity and specificity data for 50 MPXV-positive and 30 MPXV-negative clinical skin swab specimens from the 2022 MPXV outbreak. 2 μL of crude specimen from each donor was amplified in LAMP reactions designed to detect either Clade II, pan-MPXV, or pan-OPXV for 60 minutes. Additionally, composite assay sensitivity and specificity were calculated for parallel testing requiring either 2/3 or 3/3 targets to be positive.

Assay sensitivity was also benchmarked for each primer set against the 50 samples, both overall and in an approximate Ct value-stratified manner (Supplemental Table S2). Because our dataset was comprised of only clade IIb-positive samples, sensitivity was calculated as (detected positives / true positives) for n=3 replicates of each sample with each primer set. The clade II-specific assay demonstrated an overall sensitivity of 94 ± 3%, with 100% sensitivity for low Ct samples (Ct~20-25), 97 ± 2% for Ct~30, and 67 ± 17% for Ct~35 samples. Despite the drop off in sensitivity for the high Ct / low viral load samples, no sample failed to be detected in all 3 replicates in the clade II-specific— or any other— assay. The overall sensitivity for the pan-MPXV and pan-OPXV primer sets were determined as 92 ± 0 % and 93 ± 4%, respectively. Ct-stratified sensitivity followed a similar trend as for the clade II-specific assay, with the pan-MPXV and pan-OPXV assays yielding (100 ± 0 / 100 ± 0 / 100 ± 0 / 85 ± 0 %) and (100 ± 0 / 97 ± 3 / 96 ± 5 / 67 ± 7 %) for the Ct20, 25, 30, and 35 benchmark samples, respectively. Overall, the pan-MPXV primer set yielded the highest sensitivity and least variation between sample replicates, with 100% detection for all but the highest Ct value specimens. Composite sensitivity and specificity for 2/3 and 3/3 parallel targets was calculated from qPCR-benchmarked positive and negative lesion swab samples to be 99%/90% and 85%/100% for the 2/3 and 3/3 conditions, respectively (Figure 5). In practice, a test matrix with clade II-positive and pan-MPXV-negative or clade II-positive and pan-OPXV-negative result would necessitate sample retesting and sequencing in a clinical lab.

### Quantification of Nonspecific Amplification via Nanopore Sequencing

**Figure 6.**
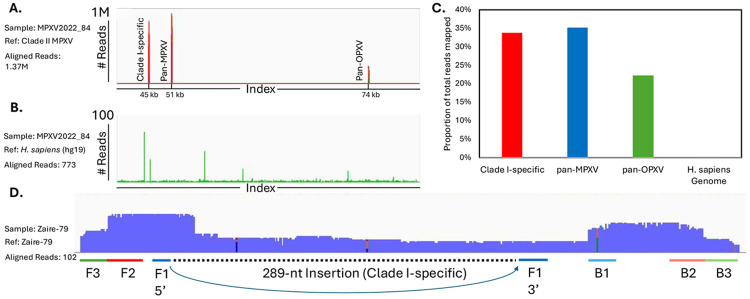
Nanopore sequencing of patient sample derived clade II-specific, pan-MPXV, and pan-OPXV amplicons aligned to clade II viral genomic DNA. **(A)** LAMP amplicon reads from clade II, pan-MPXV, and pan-OPXV primer sets pooled and aligned to MPXV2022 reference sequence and, **(B)** background amplification of human genomic DNA from the same reactions. **(C)** Total proportion of reads mapped for each primer set for patient sample template. **(D)** Clade-II specific primer set background amplification of a clade I template- Zaire-79 gDNA. Annotations denote initial primer binding regions (F2/B2) and disruption of barbell-forming F1c by a 289 bp insertion.

Since LAMP assays frequently have nonspecific background amplification, we quantified both specific and non-specific products from each assay on clade I and II templates *via* nanopore sequencing. We amplified each of our three assays on a patient sample with high viral load, pooled the resulting amplification products, and subjected them to library preparation and sequencing. An alignment of sequenced strands to both clade II template and background human genome hg19 demonstrates the specificity of the designs—of approximately 1.5M collected reads, 1.37M aligned to target regions (Figure 6A) and only 773 aligned anywhere on the human genome (Figure 6B). The assays amplified with similar efficiencies, with 34%, 35%, and 22% of reads mapping to the clade II, pan-MPXV, and pan-OPXV target regions, respectively (Figure 6C). We also tested whether the clade II-specific primers would amplify in the presence of clade I template for 90 minutes. The resulting sequencing products were aligned to a Clade I reference sequence (Zaire-79) with a total of 102 reads mapped to the insertion-bridged target region (Figure 6D). The few reads that mapped included extended inner and outer primers, with four total reads spanning the 289-nucleotide insertion specific to clade I.

### Implementation of Assays in Microfluidic Cartridge Point-of-Care Format

**Figure 7.**
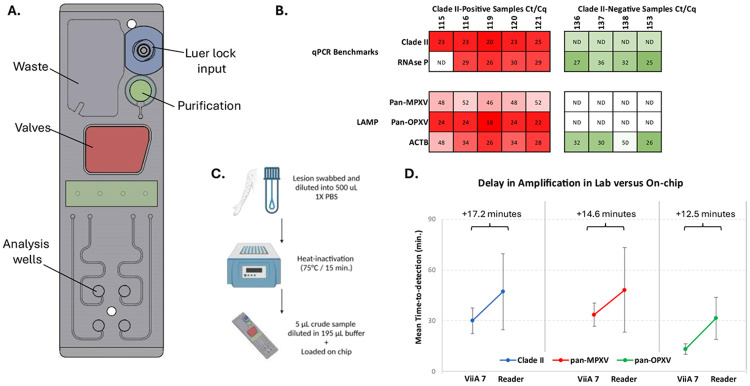
Fully on-chip validation of MPXV lesion swab samples. **(A)** Schematic diagram of 4 channel microfluidic assay cartridge with integrated sample purification Channels on top and bottom side of cartridge (top side shown). **(B)** Time-to-detection heat map for five MPXV2022 clinical samples purified and amplified completely on chip, with four negative control healthy donor skin swabs. All samples amplified for 60 minutes. **(C)** Workflow for testing crude patient samples on chip. **(D)** Relative speed performance between lab-based and on-chip amplification times for each LAMP assay showing a fairly consistent delay of ~10 minutes.

We next tested lesion swab samples in a microfluidic cartridge-based format, for which the hardware is described in prior publications^27,28,29^. Five μL of crude lysate was spiked into 195 μL silica binding buffer and loaded *via* syringe onto the chip (Figure 7A-B). Samples were bound inline to silica beads and washed once before diverting the eluate flow to four reaction microwells containing lyophilized primer and LAMP master mix pellets. We tested five positive patient samples and four negative control skin swab samples. Within 60 minutes, all 5 positive samples were detected by clade II, pan-MPXV, and pan-OPXV primer sets (Figure 7B). False positive detection with clade II and pan-MPXV primer sets did not occur in any negative control skin swab sample^30^. In the microfluidic format we observed a delay period of approximately 15 minutes relative to the lab-based instrumentation in addition to increased variance in amplification time (Figure 7D).

Lastly, the pan-OPXV assay was compared to genomes collected from 13 non-MPXV OPXVs and tested against OPXV-containing lysates. LAMP assays used 2μL of crude lysate input as above, and of the 8 OPXVs with less than 10 SNPs present in primer-binding regions, 6 (75%) amplified before the cutoff time (Table 2). Overall, 6 of 13 poxviruses tested yielded a detectable result. Detected OPXVs include ‘Old World’ OPXVs including Vaccinia virus, representatives from three clades of Cowpox (Fin2000, Ger98, and Brighton Red), Akhmeta virus, Variola virus and Ectromelia virus. Overall, 8/9 ‘Old World’ OPXV species tested were detected (including MPXV). Taterapox virus was not detected and had only one predicted SNP in the target region. Non-OPXVs (2: deerpox virus, NY_014 poxvirus), North American OPXVs (raccoonpox virus, skunkpox virus and volepox virus) and Borealpox virus had more than 10 SNPs and failed to be detected.

## Discussion

In this study we developed and validated 3 LAMP assays for the molecular discrimination of clade II from clade I MPXV and pan-MPXV from broader OPXV lineages. These clade-specific assays use genomic rearrangement-bridging LAMP as an effective approach for distinguishing between highly (99%) homologous pathogen clades and represents a strategy based on exploitation of LAMP’s multiple target regions and the kinetic limitations of Bst2.0 DNAP^26^. These results demonstrate Bridging-LAMP to be a versatile tool for discrimination of highly homologous pathogens in low resource settings. OPXV-positive results were generated in as little as ten minutes and clade-specific results in under an hour, and remain functional in a microfluidic, portable format. In comparison with qPCR where an equivalent Ct value necessitates time for enzyme activation and cycles of denaturation, annealing, and extension, isothermal techniques can offer point-of-care results between 2 and 10 times as fast.

The lab-based and microfluidic cartridge-based format used very small input quantities (1.25-2 μL of crude swab eluate per reaction) and required no upstream sample processing other than lesion swabbing, elution into PBS, and heat-inactivation. In a microfluidic format, 5 μL of crude input sample yield results in less than 90 minutes. All reaction components are amenable to lyophilization and stable without the use of a cold chain, however only the on-chip results from this study utilized lyophilized reagent pellets. Although the cartridge-based assays consistently yield signals approximately 15-20 minutes slower than LAMP performed in the lab, the lack of thermocycling still makes these assays competitive with qPCR. Additionally, our observations of the microfluidic workflow showed that rehydration and solubilization of lyophilized reagent pellets in the cartridge microwells were likely responsible for the observed delays in amplification time (~15 minutes).

At least 20 nucleic acid-based MPXV detection assays have been developed, with three qPCR assays currently recommended by the WHO^4,16-19,31-35^. While some versions of fluorescent probe-based qPCR have the mismatch sensitivity required to differentiate between MPXV clades and OPXVs, thermocycling-based methods are less amenable to immediate outbreak response and wildlife surveillance due to the upstream purification and complex instrumentation required. Isothermal assays for MPXV are currently limited and require either downstream enzymatic methods^19^ for clade differentiation or rely on expensive, proprietary reagents^17^. The consequence of this diagnostic gap is slower results in the field and less efficient usage of resources in resource-poor regions which, as noted by the fifth meeting of the International Health Regulation (IHR) Emergency Committee on the Multi-Country Outbreak of Mpox, is principally encountered in the African regions most at risk of zoonotic MPXV outbreaks^36^.

Although specific fluorescent probe-based LAMP methods have been developed, the increase to cost and reduced signal intensity of a specific hybridization probes minimizes the strengths of LAMP in POC settings: ease of operation, low cost, and instrumentation-free detection. Another method to increase LAMP specificity is the use of Cas12b-mediated collateral cleavage of quenched reporter probes (DETECTR)^24^. While effective in side-stepping issues with non-specific amplification, these methods reduce the number of viable LAMP primer sets by necessitating a viable protospacer/gRNA within the LAMP amplicon.

Here we capitalize on the simplest form of LAMP design— intercalating dye-based detection. Thus, we chose to target genomic rearrangements within the MPXV genome in lieu of nucleotide substitutions. To avoid nonspecific amplification— a pervasive issue in isothermal amplification assays— we used secondary structure prediction tools and a custom course-grained simulation of isothermal amplification (Knappenberger, M., Anderson, K.S., unpublished). However, maintaining specificity of 90% required multiplexed assay detection (Fig.5).

Although disruption of Forward and Backward Inner Primers (FIP/BIP) (Figure S1- **green box**) binding plays a role in the differentiation between MPXV and OPXV in our clade II assay, we propose that the primary mechanism underlying the observed clade specificity is a 289-nucleotide insertion absent in the F1 regions of exclusively clade II strains. Initially, we were unsure if disruption of F1 would have an appreciable effect on amplification kinetics because no primers bind to F1 prior to the formation of the barbell template molecule. Thus, binding of FIP/BIP and displacement of FIP/BIP extension products by F3/B3 still takes place allowing some appreciable amount of linear amplification which we observe in the linear background data characterizing the clade II assay. Nanopore sequencing of clade II extension products amplified in the presence of clade I template, however, suggests that extension of primers spanning the insertion is a rare event, perhaps exceeding the processivity of *Bst* DNA Polymerase in the reaction context. Indeed, many of the mapped reads appeared to be partial “aborted” extension products. Aborted products fail to serve as exponential amplification templates except *via* the formation of chimeric molecules from opposite-oriented and overlapping aborts. Such a mechanism for nonspecific signal generation is similar to a traditional PCR assay subject to insufficient extension time. In isothermal LAMP, however, we suspect that the lack of specific melting and annealing steps renders such an intermolecular interaction thermodynamically unlikely due to aborted product secondary structure.

The final genomic element we chose to target was a 13-nucleotide insertion present in exclusively MPXV lineages (Figure S2 and S3). Our pan-MPXV assay spans this insertion with the F3 primer region, and of the New World OPXVs we included in our alignment only Alaskapox had partial homology. Specificity challenge of our clade II-specific and pan-MPXV assays with non-MPXV OPXV lysates demonstrated NSA-resistance, with non-MPXV OPXVs requiring double the amplification time as MPXV template (Clade II; 21.9 vs 35.3 minutes, Pan-MPXV; 20.0 vs 37.5 minutes) demanding a strict amplification positivity cutoff for environmental samples.

In addition to specific clade differentiation, we propose our assays as effective tools for viral surveillance of zoonotic reservoirs and smallpox as a biothreat agent. Although our OPXV assay detected only 6/13 non-MPXV OPXVs tested, the ability to utilize crude sample inputs may still lower resource requirements for viral surveillance and inform further testing regimes. Most OPXVs are known to have poorly defined tropism. This gap in knowledge makes endemic OPXV control more challenging due to an inability to define vectors and trace outbreak causes. As we have demonstrated in this study, LAMP assays are well suited to crude specimens and research by other groups has demonstrated detection of pathogens in diverse sample types with minimal purification including urine, feces, saliva, and blood^23,34,35^. To realize both the goals of POC MPXV detection and OPXV wildlife/biothreat surveillance we tested our assays on a 4-channel microfluidic cartridge with inline nucleic acid purification and waste disposal. Our tests were conducted on a 5 μL input of crude lesion swab eluate diluted 1:200 in silica binding buffer. The presence of guanidinium hydrochloride and in-line wash step incorporated into the cartridge design we expect to allow direct input of biofluids. Sampling approaches for MPXV diagnosis have traditionally included skin (scab or vesicle roof) biopsies from vesicular lesions or vesicular fluid swabs^4^. Quantitative PCR quantification of viral shedding from vesicular lesions typically indicates high viral loads^39^. In the 2022 outbreak, saliva, rectal and nasopharyngeal swabs, semen, urine, and feces have all been shown to be modes of viral shedding however the lesion swab is the most accurate specimen type for MPXV testing. These various sample mediums may represent potential targets for robust molecular diagnostics like LAMP, however, for each biofluid upstream dilution and optimization will likely need to be adjusted depending on the average concentration of polymerase inhibitors and viral load, among other considerations. All data from this study was from crude lesion swab matrix.

Our strategy represents a novel method of differentiating between highly homologous viral variants based on genomic sequence organization and potential differentiation from other rash causing diseases. Exploitation of the limitations of LAMP as a strategy to detect genomic characteristics such as order of genes or number of tandem repeats as diagnostic indicators in lieu of SNPs may find suitable applications in viral surveillance of diverse pathogens in PoC settings (Figure S4) and potential biothreats such as Variola virus (Figure S5). This approach, however, opens the door to novel diagnostic failure modes in addition to mutational accumulation (e.g. genomic rearrangements in large viruses prone to frequent recombination). Our study emphasizes the practicality of LAMP in crude sample detection scenarios when appropriate measures are taken to characterize and combat non-specific amplification. Future studies building on these strategies will depend upon accurate characterization of the limitations of isothermal DNA polymerases and the conditions under which they can be exploited.

## Methods

### Identification of Targetable Genomic Rearrangements for MPXV Discrimination

Target sites for LAMP primer design were identified via alignment of the complete genomes from seven MPXV strains (four clade I, three clade II) and ten non-MPXV OPXVs. Three significant genomic rearrangements were manually identified and categorized as specific either to WA-MPXV or pan-MPXV depending on their presence in both clades of MPXV. Gaps were removed and flanking sequences were specified as either F3, F1, B1, or B3 target sequence in PrimerExplorerv5 (https://primerexplorer.jp/e/v5_manual/index.html) and NEB’s LAMP Primer Design tool (https://lamp.neb.com/) utilized to design matching primers.

### LAMP Primer Design and *in silico* non-specific amplification predictions

Primer sets specific for (1) clade II MPXV, (2) pan-MPXV, or (3) pan-OPXV were analyzed *in silico* for secondary structure stability and primer dimer formation at 65 °C using an automated selenium webdriver script. Queried services included Nupack, Thermofisher Primer Analyzer, RNAfold, 3dRNA/DNA, and OligoEvaluator. Primer sets with predicted primer heterodimers with a free energy of < −4.0 kcal/mol at 65 °C were ruled out. Predicted LAMP amplicons were predicted *in silico* by aligning primers to a WA-MPXV reference, WRAIR 7-61. Amplicon secondary structure was predicted *in silico* using the RNAfold webserver and amplicons with significant structuring of the F1-B1 region were excluded.

### Generation of MPXV vero green monkey cell lysates for specificity and LLOD studies

To prepare MPXV crude viral stocks, BSC40 cells were initially seeded into 300cm2 flasks containing DMEM 5% FBS and left overnight until they reached 50% confluency. These cells were then infected with MPXV WRAIR 7-61 at an MOI of 0.01 for one hour, followed by further incubation with DMEM 5% FBS. After three days, the infected cells were scraped in media and centrifuged at 1,000xg for 10 minutes to form a pellet. This pellet was resuspended in 10mM Tris pH 9.0 and subsequently frozen at −80C. The frozen cell suspension underwent three cycles of freeze-thawing on ice, followed by centrifugation at 1,000xg for 10 minutes. The resulting supernatant was collected, divided into smaller aliquots, and assessed for viral concentration using BSC40 cells.

For the extraction of Monkeypox viral genomic DNA, a phenol-chloroform DNA extraction method was employed. Initially, 10^7^ PFU of MPXV viral stock were diluted with 10mM Tris pH 9.0 to a volume of 100uL. This diluted stock was then combined with an equal volume of phenol that had been saturated with 10mM tris pH 8.0 buffer. The mixture was gently inverted and subsequently centrifuged at 10,000xg for 3 minutes at room temperature. The resulting aqueous supernatant was collected and mixed with a 1:1 ratio of 25:24:1 (buffer-saturated) Phenol:Chloroform:Isoamyl-alcohol. This mixture was again centrifuged at 10,000xg for 3 minutes at room temperature. To eliminate residual phenol, the aqueous phase underwent a final wash with chloroform. The DNA was then purified by precipitation in 0.3M sodium acetate 70% ethanol overnight at -20C. After centrifugation at 20,000xg at 4C for 20 minutes, the DNA pellet was washed twice with 70% ethanol. Finally, the DNA was resuspended in nuclease-free water and its concentration was determined using a Nanodrop machine.

### Lab-based LAMP Reactions

To set up LAMP reactions, fresh 10X primer mix was first prepared from individual primer stocks: 16 μL of 100 μM FIP and BIP primers, 2 μL of 100 μM F3 and B3, and 4 μL of each 100 μM loop primer for the pan-OPXV primer set was added to a final volume of 100 μL in nuclease-free H2O. Final concentrations in 1X primer mix were 1.6 μM for FIP and BIP, 2 μM for F3 and B3, and 4 μM for the loop primers when used. Reactions were setup by adding 10 μL of 2X Warm-start LAMP enzyme mix (NEB #E1700L; Ipswich, MA), 2 μL of freshly prepared primer mix, 0.4 μL of 250 mM SYTO9 fluorescent dye (ThermoFisher #S34854; Waltham, MA), 6.6 μL of NF-H2O, and 1 μL of sample (crude or purified) for a total reaction volume of 20 μL.

### Limit of Detection Study

Limit of detection studies were carried out on purified WRAIR 7-61 vgDNA isolated from vero green monkey cell lysate as described above. vgDNA was diluted in NF-H2O at ratios of 1:10, 1:100, 1:1000, 1:10,000, 1:20,000, 1:40,000, and 1:80,000 and 1 μL of template spiked into 20 μL LAMP reactions. 8 replicates were performed for each dilution factor and were considered positive if all replicates reached detectable increase in fluorescent signal before the assay cutoff time of 40 minutes. Assay cutoff was determined by amplifying no template reactions for 60 minutes and specifying a point at least 10 minutes before non-specific amplification became apparent.

### Clade II Specificity Study

To test clade specificity between clade II and clade I MPXV we utilized WRAIR 7-61 purified genomic DNA and Zaire-79 crude genomic DNA, respectively. WRAIR 7-61 was purified as above. Zaire-79 lysate was prepared by infecting 4M vero monkey cells with 0.5e+8 PFU of Zaire-79 strain MPXV input virions. Cells were cultured in BSL-3 conditions for 5 days before being resuspended in 1X LD buffer (62.5 mM Tris, pH 6.8, 2% SDS, 12.5% glycerol) to lyse the cells and inactivate the virus prior to transfer to BSL-1. Under BSL-1 conditions, the lysate was found to be too viscous to work with, so it was heated at 95°C for 5 minutes prior to being spun down at 21,000xg for 5. Liquid from the top of the suspension was then diluted 1:100 in NF-H2O prior to spiking into fluorescent LAMP reactions at a volume ratio of 1:20. Final concentration of each 1X LD buffer component in LAMP reactions was: 125 μM Tris, 0.004% SDS, 0.025% glycerol.

### Patient Sample Benchmarking

80 heat-inactivated patient swab eluate samples were received from the Centers for Disease Control MPXV response team in a BSL-3 level laboratory. These samples were pre-existing and anonymized. Remainder specimens were deidentified according to CDC IRB protocol 7294. Testing activities were reviewed and approved by CDC as exempt human subjects research (See 45 C.F.R. part 46.104). Samples were transferred to a BSL-2+ setting for testing within biocontainment hoods. LAMP reactions were set up with crude specimens as follows: 10 μL of 2X Warm-start LAMP Enzyme Mix (NEB, Catalog #E1700L), 0.4 μL of 250 mM SYTO9 dye, 2 μL 10X primer mix (1.6 μM FIP/BIP primers, 0.2 μM F3/B3, and 0.4 μM LF/LB), and 5.6 μL of nuclease free H2O were combined in a mastermix for the required number of reactions and 18 μL of mastermix was pipetted into each well of a 96-well MicroAmp qPCR plate (Thermofisher, #N8010560). Prior to specimen addition, specimens were heated to 85 °C for 5 minutes followed by rapid transfer to ice for 3 minutes. Samples were spun down briefly. Finally, 2 μL of specimen was added to each sample well and samples were briefly mixed *via* pipetting. Reaction wells were sealed with MicroAmp Optical Adhesive Film (Thermofisher #4311971) and spun down in a tabletop centrifuge at 500 x G for 1 minute. Samples were transferred to a ViiA7 qPCR machine and amplified as follows: (1) heat to 65 °C and hold for 5 seconds, (2) cycle at 65 °C for 180 cycles of 20 seconds/cycle, collecting fluorescence in the SYBR channel at the end of each cycle, (3) heat to 95 °C for 5 minutes to inactivate enzyme. Time-to-detection values were calculated by dividing Ct by 3 to convert 20 second cycles to minutes. All samples were tested 3 times over the course of 3 separate days in independent experiments.

### Nanopore Sequencing and Alignment of LAMP Amplicon Reads to Reference Genomes

Nanopore library preparation was performed for pooled LAMP reactions (20 μL / reaction) for clade II, pan-MPXV, and pan-OPXV primers amplified in the presence of clade IIb template (MPXV2022-84) as instructed by the manufacturer (Oxford Nanopore, Oxford, United Kingdom). Briefly, amplicons were purified *via* ethanol purification and prepared for sequencing with the Oxford Nanopore Ligation Sequencing Kit XL V14 (#SQK-LSK114-XL). After sequencing on a MinION R9.4.1 flow cell (#FLO-MIN106D), reads were mapped using MiniMap2 to either a clade I or II reference genome, or to the human hg19 reference genome to quantify off-target amplification. Reads were filtered prior to mapping by removing nanopore adaptors with PoreChop. Concatemers were not split due to LAMP’s ability to generate concatenated sequences. For the nonspecific amplification experiment all procedures were the same except amplicons were generated by amplifying just clade II-specific primers in the presence of clade I template isolated from cultured virus-infected cells. Reads for the nonspecific amplification experiment were mapped to the clade I Zaire-96 reference genome.

### Amplification of patient samples on a microfluidic chip

The microfluidic cartridge was designed with a Luer lock to connect common syringes for sample and buffer input. For each subject, a 5 μL aliquot of the lesion sample was added to 200 μL of a binding buffer mixed from 2 M GuHCl, 10 mM Tris HCl, and 1 mM EDTA. The reaction mix was vortexed and then 200 μL of 100% ethanol was added and vortexed again. The microfluidic cartridge was placed into the valve actuator and set to divert the fluid to waste. The mixture was loaded into a 3 mL syringe (BD) and connected to the cartridge. Plunging the syringe allowed the mixture to pass through a silica membrane at the purification section of the cartridge to collect nucleic acids, and any remaining fluid went to the waste. A 1 mL syringe (Nipro) pre-loaded with 140 μL of buffer AE (Qiagen) was connected to plunge any residual binding buffer and inhibitors to waste. The valve actuator was changed to divert the fluid to the analysis wells. A second 1 mL syringe pre-loaded with 200 μL of buffer AE was plunged through the cartridge, which filled the analysis wells with eluted nucleic acids. The analysis wells were pre-loaded with lyophilized LAMP reagent beads and primers for each target sequence. The filled cartridge was placed in the reader for heating and fluorescence detection over 90 minutes.

## Supplementary Material

Supplement 1

## Figures and Tables

**Table 1.** Predicted specificity for 3 pox-specific LAMP assays designed around genomic gaps present in Clade II MPXV, Clade I MPXV, and 10 OPXV clades including *Variola major* and the non-traditional OPXVs Murmansk virus and NY_014. The Clade II-specific assay uses a FIP primer bridging a 289 bp insertion present in the stem-loop determining region (F1) not present in clade I MPXV or OPXVs. Pan-MPXV assay prevents strand-displacement of first synthesis products by outer primers *via* bridging an insertion in F3, preventing exponential amplification.

Specificity	Clade IIb	Clade IIa	Clade Ia	pan-OPXV	Variola	Sequence-specific Disruption
**Clade II**	X	X	-	-	-	Loop 2' structure disruption (F1c - 289bp gap)
**pan-MPXV**	X	X	X	-	-	Strand-displacement disruption (F3 - 27bp gap)
**pan-OPXV**	X	X	X	X	X	N/A

**Table 2.** Empirical detection of 13 non-MPXV OPXVs in Vero Green Monkey cell lysate and comparison with pan-OPXV assay specificity calculated from summing primer mismatches in each OPXV genome. 6/13 OPXVs are detected before assay cutoff.

		Genomic Target Region Mismatches/Indels							Empirical Results	
Pan-OPXV LAMP Assay		F3	F2	F1	B1	B2	B3	Total SNPs	OPXV002 Result	Mean Cq (min.)
**MPXV**	*USA2022_MA001*	0	0	0	0	0	0	**0**		
	*Nigeria-SE-1971*	0	0	0	0	0	0	**0**		
	*MPXV Refseq NC_063383.1*	0	0	0	0	0	0	**0**		
	*Zaire-96-1-16*	0	0	0	1	0	0	**1**		
	*Congo-8*	0	0	0	0	0	0	**0**		
	*Cameroon-1990*	0	0	0	0	0	0	**0**		
**Old-World**	*Vaccinia Virus*	1	1	0	0	0	0	**2**	**POS**	**17.6**
	*Cowpox, Fin2000*	0	1	0	0	0	0	**1**	**POS**	**17.8**
	*Cowpox, Ger98*	0	1	0	0	0	0	**1**	**POS**	**22**
	*Cowpox, Brighton Red*	5	1	1	1	0	0	**8**	**POS**	**12.3**
	*Taterapox*	0	1	0	0	0	0	**1**	**NEG**	**26.9**
	*Akhmeta virus*	1	3	1	3	1	0	**9**	**POS**	**23.7**
	*Borealpox*	1	2	0	1	1	2	**7**	**NEG**	**25.7**
	*Ectromelia*	5	1	0	2	0	1	**9**	**POS**	**20.7**
**New-World OPXV**	*Skunkpox WA*	3	7	3	4	1	4	**22**	**NEG**	**29.5**
	*Volepox CA*	3	6	3	4	2	4	**22**	**NEG**	**25.6**
	*Raccoonpox*	2	7	2	3	2	13	**29**	**NEG**	**31.6**
**Non-OPXV**	*Deerpox (W-848-83)*	16	6	3	6	5	17	**53**	**NEG**	**26.8**
	*NY014*	4	10	17	24	7	2	**64**	**NEG**	**26.8**

## Significance Statement

This study describes loop-mediated isothermal amplification (LAMP) assays for detection and differentiation of clade I and II *Orthopoxvirus Monkeypox* virus, as well as the broader *Orthopoxvirus* genus, using a novel LAMP-based technique— Bridging LAMP.
