# Supplementary material for: Portable Detection of Monkeypox virus Clades using Bridging LAMP
Source: Res Sq. 2026 May 12:rs.3.rs-9014850. Preprint. [Version 1] doi: 10.21203/rs.3.rs-9014850/v1 (PMC13193100; doi:10.21203/rs.3.rs-9014850/v1)
Supplement: Supplement 1 [file NIHPPrs9014850v1-supplement-1.pdf]

Supplemental Figures and Tables:

Assay	Primer Name	Sequence (5'-3')
**Clade II-specific**	MPXV_003-F3	TGATATATGCTCGCTGCTT
	MPXV_003-B3	GCCTATTCACTAACAAAGTCAA
	MPXV_003-FIP	CATGAACAATGTTGGATGCATTCTATACATGTTTCCCAATTTGTTTG
	MPXV_003-BIP	ATGTATGCCTGTTCGTATGCCTGTGCACTCTTAAAGATTCTCAAAG
**pan-MPXV**	MPXV_004-F3	CTAACTAAAAGACATAAAAAGCGG
	MPXV_004-B3	CCAAATTGCAGTTTCTGGT
	MPXV_004-FIP	CTTTGTGCATTTTTGTACACATCGTTAGGATATAAATATTATGGCCGCA
	MPXV_004-BIP	AGAGGTCTAAGTAGACCGTTAATGAATCCATCCATACGCGTAAT
**pan-OPXV**	OPXV_002-F3	GGTGGTCTTTCGGCATTA
	OPXV_002-B3	AACGTATCCCCCTGACAC
	OPXV_002-FIP	CTGATATCTTTCCATCCTTCTTCCAGACAATCATATAATGGAACCGC
	OPXV_002-BIP	AGTATGAAGAGGGTGCAGTATTAGATCGAATAAATCAACATCGCATT
	OPXV_002-LF	TGCGACAGCAATTGGGT
	OPXV_002-LB	CCGACGTCCACGACACT

Supplemental Table SI. LAMP Primers for specific for clade II MPXV, pan-MPXV, and broad New World OPXVs including VARV and MPXV. Clade II and pan-MPXV incorporate no loop primers. LAMP primer set composed of Forward Outer (F3) and Reverse Outer (B3) initial product displacement primers, Forward Inner (FIP) and Reverse Inner (BIP) turnback primers, and Loop Forward (LF) and Loop Back (LB) amplification primers.

Benchmark Ct	Clade II-specific Sensitivity + SD	pan-MPXV Sensitivity + SD	pan-OPXV Sensitivity + SD
20	100 ± 0%	100 ± 0%	100 ± 0%
25	100 ± 0%	100 ± 0%	98 ± 3%
30	98 ± 3%	100 ± 0%	96 ± 6%
35	67 ± 18%	86 ± 0%	67 ± 7%
Overall	**94 ± 4%**	**98 ± 0%**	**93 ± 4%**

Supplemental Table S2. Tabulated assay sensitivity for Clade II-specific, pan-MPXV, and pan-OPXV assays benchmarked to 50 unpurified swab eluate samples collected during the USA2022-2023 outbreak. Sensitivity was calculated as the mean of detected positives divided by total positives for 3 replicates of each sample. Benchmark Ct values are approximate and were provided with the anonymized samples.

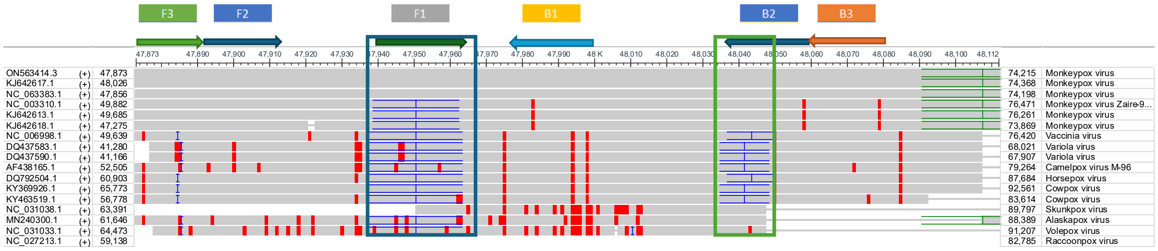

		Genomic Target Region Mismatches/Indels					
MPXV003		F3	F2	F1	B1	B2	B3
MPXV	USA2022_MA001	0	0	0	0	0	0
	Nigeria-SE-1971	0	0	0	0	0	0
	MPXV Refseq NC_063383.1	0	0	0	0	0	0
	Zaire-96-I-16	0	0	0 + 253 nt insertion	1	1	2
	Congo-8	0	0	0 + 253 nt insertion	1	1	2
	Cameroon-1990	2	0	0 + 259 nt insertion	1	1	2
OPXV	Vaccinia Virus	2	0	0 + 391 nt insertion	2	1 + 24 nt insertion	0
	Variola (Congo - 1970)	0	1	2 + 391 nt insertion	2	1 + 24 nt insertion	0
	Variola (Somalia - 1977)	7	1	2 + 391 nt insertion	2	1 + 24 nt insertion	0
	Camelpox M-96	3	3	2+ 388 nt insertion	2	1 + 24 nt insertion	1
	Horsepox MNR-76	2	0	0 + 391 nt insertion	2	1 + 24 nt insertion	0
	Cowpox (Kostroma - 2015)	2	0	0 + 391 nt insertion	2	1 + 24 nt insertion	0
	Cowpox (Germany - 1971 EP1)	2	0	0 + 391 nt insertion	2	1 + 24 nt insertion	1
	Skunkpox WA			12	9	13	
	Alaskapox (Alaska - 2015)	3	2	4 + 391 nt insertion	6	13	
	Volepox CA	7	3	4 + 385 nt insertion	9	14	
	Raccoonpox						

Indexed Target Locations			
F3	47873-47891	B3	48058-48081
F2	47892-47913	B2	48037-48059
F1	47939-47963	B1	47977-47999

Supplementary Figure S1. Clade II Specificity Design. Alignment of MPXV and New World OPXVs with Congo Basin/OPXV-specific translocation bounded by F1 primer. Green box denotes insertion allowing BIP-mediated specificity for MPXV but not OPXV. Blue box denotes clade II-specific insertion. We suggest potential mechanisms underlying our observed assay specificity depending on the positioning of the target genomic rearrangement relative to the primer binding sites. Each mechanism disrupts formation of the canonical double stem-loop exponential amplification 'barbell' that serves as template for the second phase of the reaction at a different time point**.** The first mechanism is disruption of initial FIP/BIP primer binding *via* bridging the F2 or B2 region (e.g. MPXV-specific 27 nucleotide insertion targeted by pan-MPXV assay). As formation of active 'dumbell' templates is first and foremost reliant upon specific extension of FIP/BIP primers, extension of upstream F3 and B3 strand-displacement primers in the absence of F1c/B1c overhangs render the inner and exponential loop primers LF/LB inert[22,37]. Our findings suggest that extension and self-displacement of F3/B3 by Bst2.0 DNA polymerase is insufficient to generate an observable SYTO9 signal over the course of 60 minutes. This failure to achieve efficient linear amplification can likely be attributed to thermodynamic limitations on strand-displacement synthesis to sub-kilobase lengths by Bst polymerase in contrast with phage DNA polymerases such as phi29, as well as a low efficiency of F3/B3 auto-catalytic self-displacement [26,38]. Additionally, the low concentration of F3/B3 in the LAMP reaction (200 nM each) positions these primers as the limiting reagent in most conceivable F3/B3-mediated linear amplification mechanisms.

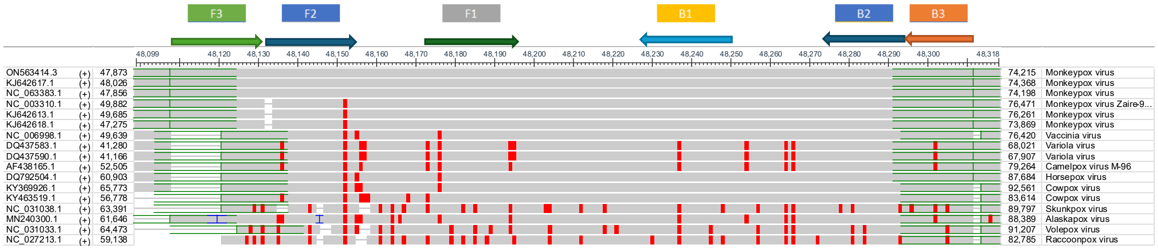

		Genomic Target Region Mismatches/Indels					
MPXV004		F3	F2	F1	B1	B2	B3
MPXV	USA2022_MA001	0	0	0	0	0	0
	Nigeria-SE-1971	0	0	0	0	0	0
	MPXV Refseq NC_063383.1	0	0	0	0	0	0
	Zaire-96-I-16	0	3	0	0	0	0
	Congo-8	0	3	0	0	0	0
	Cameroon-1990	0	3	0	0	0	0
OPXV	Vaccinia Virus	13	2	1	0	0	0
	Variola (Congo - 1970)	13	2	4	1	0	1
	Variola (Somalia - 1977)	13	2	4	1	0	1
	Camelpox M-96	13	2	3	1	0	1
	Horsepox MNR-76	13	2	1	0	0	0
	Cowpox (Kostroma - 2015)	13	2	1	0	0	0
	Cowpox (Germany - 1971 EP1)	13	2	1	0	0	0
	Skunkpox WA	13	6	4	2	3	4
	Alaskapox (Alaska - 2015)	0 + 5 nt insertion	5	2	1	0	1
	Volepox CA	13	7	4	2	2	1
	Raccoonpox	13	6	6	4	3	2

Indexed Target Locations			
F3	48108-48131	B3	48293-48311
F2	48132-48155	B2	48274-48294
F1	48172-48196	B1	48228-48250

Supplementary Figure S2. Pan-MPXV Specificity Design. Alignment of MPXV and New World OPXVs with 5’ deletion in OPXV lineages predicted to yield a pan-MPXV-specific assay.

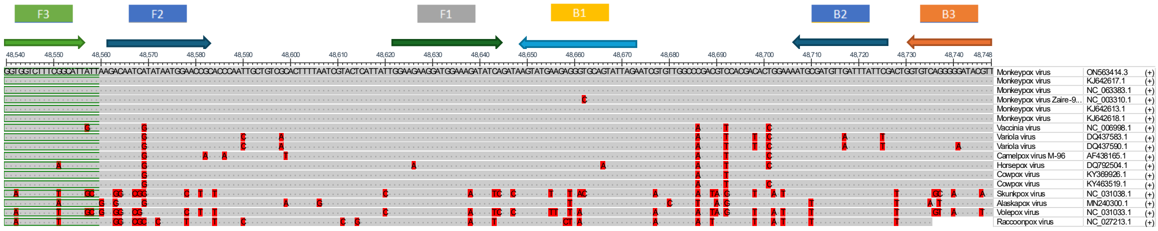

		Genomic Target Region Mismatches/Indels					
OPXV002		F3	F2	F1	B1	B2	B3
MPXV	USA2022_MA001	0	0	0	0	0	0
	Nigeria-SE-1971	0	0	0	0	0	0
	MPXV Refseq NC_063383.1	0	0	0	0	0	0
	Zaire-96-I-16	0	0	0	1	0	0
	Congo-8	0	0	0	0	0	0
	Cameroon-1990	0	0	0	0	0	0
OPXV	Vaccinia Virus	1	1	0	0	0	0
	Variola (Congo - 1970)	0	1	0	0	2	0
	Variola (Somalia - 1977)	0	1	0	0	2	1
	Camelpox M-96	0	2	0	0	0	0
	Horsepox MNR-76	1	1	1	1	0	0
	Cowpox (Kostroma - 2015)	0	1	0	0	0	0
	Cowpox (Germany - 1971 EP1)	0	1	0	0	0	0
	Skunkpox WA	3	7	3	4	1	4
	Alaskapox (Alaska - 2015)	1	2	0	1	2	2
	Volepox CA	3	6	3	4	2	4
	Raccoonpox	2	7	2	3	2	13

Indexed Target Locations			
F3	48540-48557	B3	48731-48748
F2	48562-48583	B2	48706-48727
F1	48621-48645	B1	48649-48673

Supplementary Figure S3. Pan-OPXV Specificity Design. Alignment of MPXV and New World OPXVs demonstrating limited mismatches across the OPXV primer targets.

Specimen Information				Clade II-Specific		pan-MPXV		pan-OPXV	
Panel ID	Sample Type	Suspected Disease	Clade II qPCR ~Ct	Cq + SD (min)	Ct-Stratified Cq mean + SD (min)	Cq + SD (min)	Ct-Stratified Cq mean + SD (min)	Cq + SD (min)	Ct-Stratified Cq mean + SD (min)
MPX2022-84				26.8 ± 1		23.2 ± 0.8		10.2 ± 0.1	
MPX2022-86				28.6 ± 1.1		24.8 ± 0.9		11 ± 0.1	
MPX2022-88				28.5 ± 1.6		24.4 ± 1.1		10.6 ± 0.2	
MPX2022-95	Swab Eluate (unpurified)	Mpox	20	28.5 ± 0.9	27.9 ± 0.9	24.7 ± 1.1	23.9 ± 0.8	10.7 ± 0.1	10.6 ± 0.3
MPX2022-103				28 ± 1.8		23.1 ± 2.8		10.5 ± 0.8	
MPX2022-108				28.5 ± 1.5		24.4 ± 0.9		10.6 ± 0.1	
MPX2022-110				26.5 ± 0.4		22.9 ± 0.4		10.2 ± 0.3	
MPX2022-73				35.8 ± 4.1		31.3 ± 3		14.2 ± 2.3	
MPX2022-77				31.3 ± 0.8		28.1 ± 1.2		12.2 ± 0.4	
MPX2022-78				37 ± 2.1		34.2 ± 1.6		14.6 ± 0.4	
MPX2022-85				30.6 ± 0.8		27.2 ± 1.2		11.7 ± 0.1	
MPX2022-87				36.9 ± 1.3		33.2 ± 1.8		14 ± 0.2	
MPX2022-90				35.7 ± 2.4		31.1 ± 1.4		13.8 ± 0.6	
MPX2022-92				33.4 ± 1		28.9 ± 0.9		13 ± 0.5	
MPX2022-93	Swab Eluate (unpurified)	Mpox	25	31 ± 1.3	32.9 ± 2.8	27.2 ± 1.5	29 ± 2.7	12 ± 0.1	12.7 ± 1.2
MPX2022-96				35.9 ± 1.6		31.3 ± 1.7		13.3 ± 0.4	
MPX2022-101				33.7 ± 2.8		29.3 ± 1.3		14.2 ± 0.8	
MPX2022-102				30.6 ± 1.6		27.5 ± 1.3		11.7 ± 0.3	
MPX2022-105				31.8 ± 1.1		28.3 ± 0.8		12.1 ± 0.3	
MPX2022-106				28.6 ± 1.3		25.1 ± 1.2		10.9 ± 0.2	
MPX2022-107				29.2 ± 1.3		25.2 ± 0.7		10.8 ± 0.1	
MPX2022-109				32.3 ± 1.1		27.5 ± 1.1		11.8 ± 0.1	
MPX2022-70				37.6 ± 4.1		35 ± 1.5		14.9 ± 0.3	
MPX2022-72				38.2 ± 0.9		34.3 ± 1.5		16.3 ± 2.5	
MPX2022-75				ND		40.5 ± 2.1		15.7 ± 0.5	
MPX2022-76				40.4 ± 3		37.8 ± 1.3		16.8 ± 1.3	
MPX2022-80				39.1 ± 2.1		35.2 ± 2		14.7 ± 0	
MPX2022-81				40.4 ± 0.5		36.8 ± 0.7		14.8 ± 1	
MPX2022-82				37 ± 1.7		32.9 ± 1.5		13.5 ± 0.8	
MPX2022-83	Swab Eluate (unpurified)	Mpox	30	38.2 ± 0.8	39.5 ± 2.9	33.5 ± 1	35.8 ± 2.8	14.1 ± 0.3	15.9 ± 2.8
MPX2022-89				44 ± 4.2		37.3 ± 0.3		16.5 ± 1.3	
MPX2022-91				44.8 ± 1.2		39.4 ± 2.6		15.6 ± 0.6	
MPX2022-97				37.5 ± 1.9		32.9 ± 2.2		14.3 ± 0.5	
MPX2022-99				41.4 ± 3.5		36.4 ± 1.9		16.4 ± 1.6	
MPX2022-100				35.2 ± 1.9		32 ± 1.2		13.8 ± 0.2	
MPX2022-111				35.4 ± 0.9		31.9 ± 1.5		13.8 ± 1.1	
MPX2022-112				40.8 ± 2.8		39.7 ± 2.7		25.1 ± 14	
MPX2022-114				42.1 ± 5.4		36.7 ± 2.1		17.7 ± 3.9	
MPX2022-71				50.2 ± 2.5		41.5 ± 3.6		18.6 ± 1.9	
MPX2022-74				46.7 ± 4.3		47 ± 4.5		16.9 ± 0	
MPX2022-79				44 ± 6.4		40.5 ± 2.4		17 ± 0.5	
MPX2022-94	Swab Eluate (unpurified)	Mpox	35	48.7 ± 0	46.7 ± 2.4	45.9 ± 4.4	44 ± 1.9	18.4 ± 1.2	19.2 ± 2.3
MPX2022-98				41.4 ± 0.4		41.2 ± 0.3		17.9 ± 2.1	
MPX2022-104				54.3 ± 6.4		51.6 ± 0		23.3 ± 6.7	
MPX2022-113				41.5 ± 0		40.6 ± 5.9		22 ± 0	

Panel ID	Type	Disease	qPCR Ct	Clade II-Specific	pan-MPXV	pan-OPXV
MPX2022-125				ND	ND	28.5 ± 3.4
MPX2022-126				ND	37.5 ± 5.5	31.3 ± 1.8
MPX2022-127				ND	38.5 ± 0	31.8 ± 1.1
MPX2022-128				40.6 ± 3.5	ND	31.4 ± 0.3
MPX2022-129				ND	ND	32.6 ± 3.1
MPX2022-130				ND	ND	31.6 ± 0.6
MPX2022-131				34.4 ± 0	ND	32.7 ± 0.6
MPX2022-132				ND	ND	31 ± 1.8
MPX2022-133				ND	ND	34.6 ± 0.4
MPX2022-134				ND	ND	ND
MPX2022-135				ND	ND	31.2 ± 6.6
MPX2022-136				ND	ND	ND
MPX2022-137				31.6 ± 0.2	36.2 ± 8.6	29.3 ± 1.8
MPX2022-138				ND	ND	35.1 ± 2.4
MPX2022-139	Swab Eluate (unpurified)	Non-MPXV	ND	ND	ND	28 ± 1.6
MPX2022-140				ND	ND	34.6 ± 1.6
MPX2022-141				ND	ND	37.9 ± 7.5
MPX2022-142				30.8 ± 0	33.1 ± 2.1	26.5 ± 3.3
MPX2022-143				ND	ND	27.7 ± 1.2
MPX2022-144				ND	ND	32.6 ± 10.9
MPX2022-145				ND	ND	26.1 ± 3.9
MPX2022-146				34.6 ± 3.1	34.5 ± 1.8	28.6 ± 0.4
MPX2022-147				29.1 ± 0	ND	26.1 ± 2
MPX2022-148				21.3 ± 0.2	21.6 ± 0.7	22.2 ± 0.5
MPX2022-149				44.7 ± 0	ND	32 ± 3.4
MPX2022-150				46.2 ± 0	35.3 ± 4.1	27.7 ± 0.2
MPX2022-151				26.5 ± 0	ND	27.4 ± 0.8
MPX2022-152				35.5 ± 0	ND	28.1 ± 3.1
MPX2022-153				ND	ND	29.2 ± 5.2
MPX2022-154				32.5 ± 5.6	ND	32.7 ± 0.2

Supplemental Table S3. Detection of crude patient specimens collected during the 2022 MPXV outbreak. Tabulated results (n=3) for unpurified swab eluate samples on 3 LAMP primer sets: (1) clade II-specific, (2) pan-MPXV, and (3) pan-OPXV. All sample time-to-detection values were calculated as the mean of 3 independent replicates from 3 experiments on 3 separate days plus standard deviation of the mean. Anonymized samples were provided with stratified, approximate benchmark Ct values, so Ct-stratified means were calculated by grouping samples accordingly.

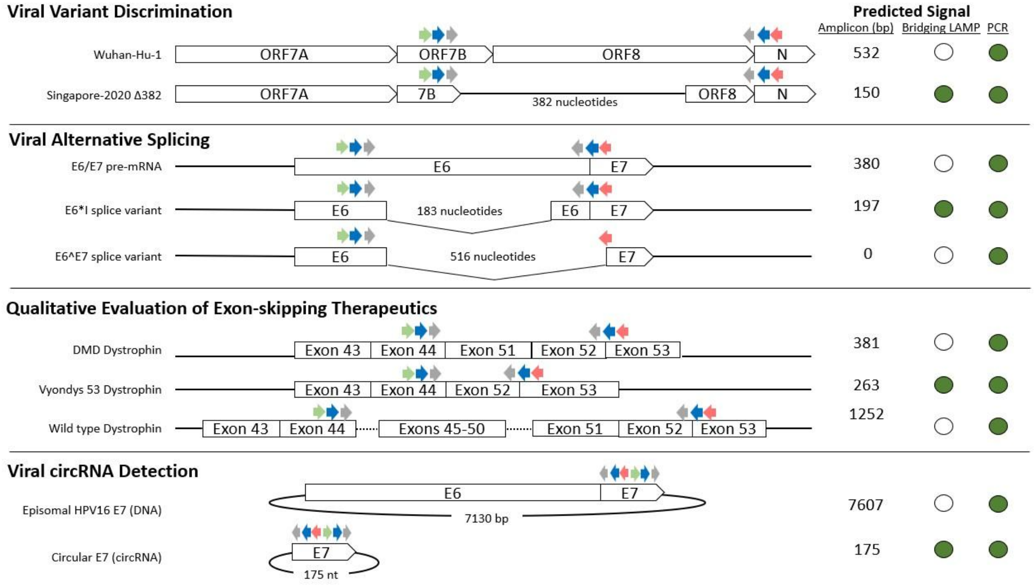

Supplemental Figure S4. Generalized application of bridging LAMP strategies for rapid detection of clinically relevant biomarkers. Bridging LAMP amplification predicted based on an amplicon size less than 250-300 nucleotides. PCR amplification prediction based on assumption of sufficient extension time to synthesize full amplicon. **(A)** Discrimination of wild type SARS-CoV-2 from the clinically milder Δ382 deletion variant *via* bridging of ORF7b/ORF8. **(B)** Detection of the HPV16 E6*I splice variant associated with cervical disease progression without detection of E6/E7 pre-mRNA or E6^E7 chimeric splice variant. **(C)** Specific detection of dystrophin Exon 43-44-52-53 resulting from treatment of Duschenne muscular dystrophy (DMD) dystrophin with exon skipping therapeutic antisense oligonucleotide. Retention of exon 51 in DMD dystrophin raises amplicon length above 300 nucleotides, severely reducing efficiency of the LAMP reaction. **(D)** Outward facing bridging LAMP strategy for detection of clinically severe HPV16 E7 backspliced circRNA variant in comparison with episomal HPV16 DNA.

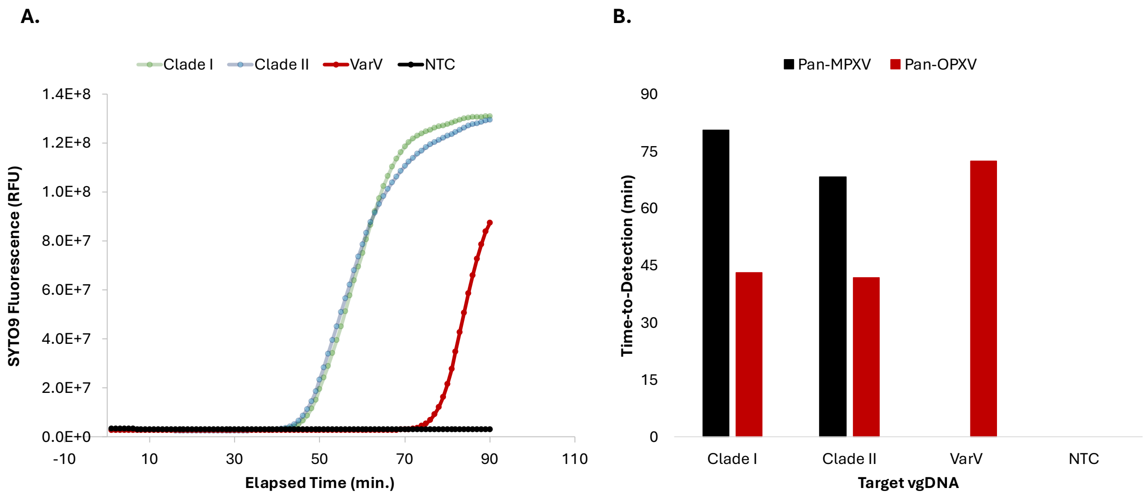

Supplemental Figure S5. Detection of Variola vgDNA by pan-OPXV LAMP assay. **(A)** Real-time fluorescence amplification curves of Clade I, Clade II MPXV, VARV, or NTC amplified with pan-OPXV primers for 90 minutes showing detection of all three targets with a time-delayed VARV response. **(B)** Time-to-Detection for Clade I, Clade II MPXV, and VARV with pan-MPXV and pan-OPXV LAMP assays showing detecting of VARV is specific to the pan-OPXV primer set.

Limit of Detection

Replicates amplified per serial dilution (WRAIR 7-61)

Primer Set	6500 copies	650 copies	325 copies	163 copies	81 copies	65 copies	0 copies (NTC)
Clade II-specific	10/10 (100%)	7/7 (100%)	7/7 (100%)	5/7 (71%)	1/7 (14%)	0/7 (0%)	0/10
Pan-MPXV	7/7 (100%)	7/7 (100%)	3/3 (100%)	3/3 (100%)	3/3 (100%)	1/4 (25%)	0/20
Pan-OPXV	7/7 (100%)	7/7 (100%)	3/3 (100%)	3/3 (100%)	2/3 (66%)	1/4 (25%)	0/20

Supplementary Figure S6. LLOD table for each LAMP assay vs. Clade II WRAIR 7-61 purified genome.

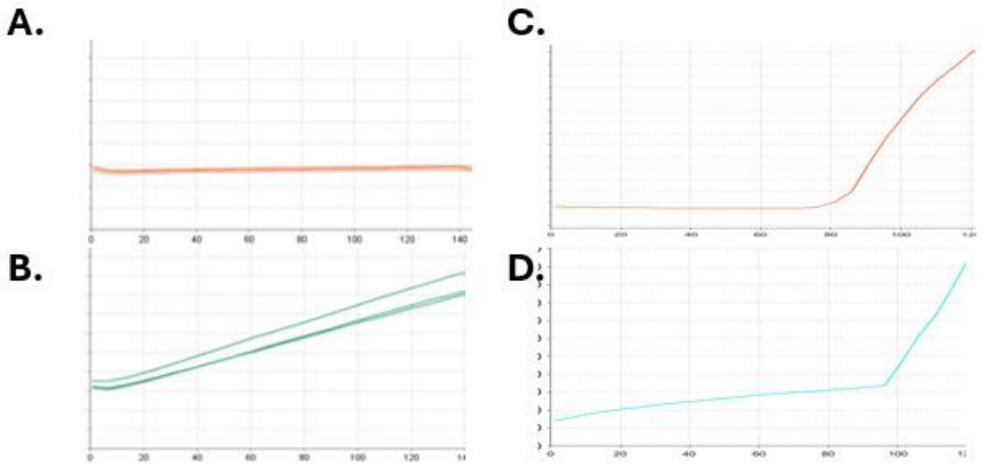

Supplementary Figure S7. (A-D) Different categories of background amplification common to different LAMP primer sets (A) No background, (B) linear background, (C) log background, and (D) lin-log background. Primer sets categorized as no background or linear background were moved forward to testing for testing of specific MPXV template.

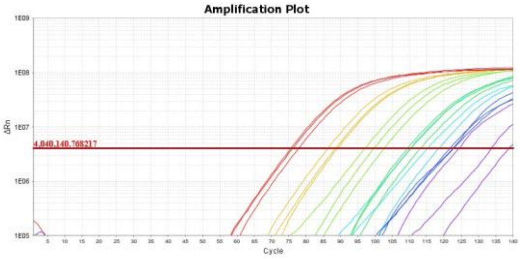

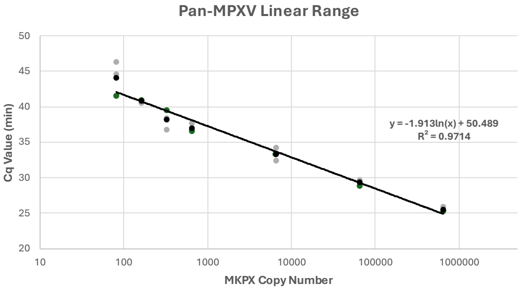

[MPXV Template]	Dilution Factor
6.5e+5 cps/rx (3.25e+4 cps/uL)	1:10
6.5e+4 cps/rx (3.25e+3 cps/uL)	1:100
6500 cps/rx (325 cps/uL)	1:1000
650 cps/rx (32.5 cps/uL)	1:10,000
325 cps/rx (16.25 cps/uL)	1:20,000
162.5 cps/rx (8.1 cps/uL)	1:40,000
81.3 cps/rx (4.1 cps/uL)	1:80,000
No cps/rx (-ve control)	1:inf.

Supplementary Figure S8. Pan-MPXV LAMP Assay LLOD Raw fluorescence curves with Clade II template.

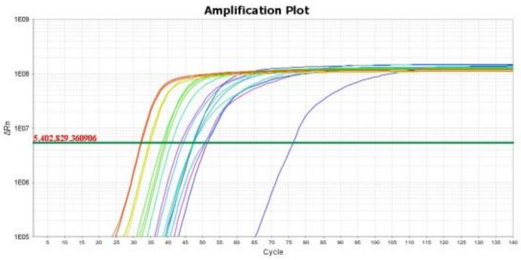

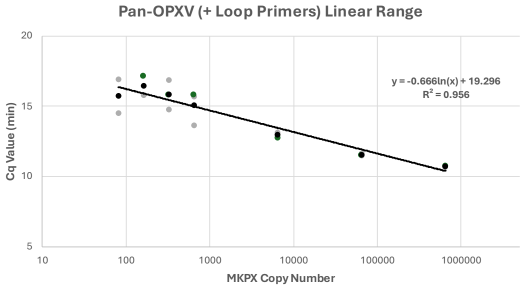

[MPXV Template]	Dilution Factor
6.5e+5 cps/rx (3.25e+4 cps/uL)	1:10
6.5e+4 cps/rx (3.25e+3 cps/uL)	1:100
6500 cps/rx (325 cps/uL)	1:1000
650 cps/rx (32.5 cps/uL)	1:10,000
325 cps/rx (16.25 cps/uL)	1:20,000
162.5 cps/rx (8.1 cps/uL)	1:40,000
81.3 cps/rx (4.1 cps/uL)	1:80,000
No cps/rx (-ve control)	1:inf.

Supplementary Figure S9. Pan-OPXV LAMP assay LLODs against Clade II template.
